# Characterizing Response to PARP Inhibitor Treatment Combinations in Advanced Prostate Cancer

**DOI:** 10.3390/biomedicines14050949

**Published:** 2026-04-22

**Authors:** Bryan Correa Gonzalez, Akshaya Karthikeyan, Love A. Moore, Anamitra Bhaumik, Ethan Sandoval, Marion Hardy, John D. McPherson, Hong Li, Mamta Parikh, Marc Dall’Era, Allen C. Gao, Alan P. Lombard

**Affiliations:** 1 Department of Urologic Surgery, University of California, Davis, Sacramento, CA 95817, USA; bcorreagonzalez@health.ucdavis.edu (B.C.G.); akarthik@health.ucdavis.edu (A.K.); mdallera@health.ucdavis.edu (M.D.); acgao@health.ucdavis.edu (A.C.G.); 2 Department of Molecular and Cellular Biology, University of California, Davis, Davis, CA 95616, USA; mhardy@ucdavis.edu; 3 Department of Biochemistry and Molecular Medicine, University of California, Davis, Sacramento, CA 95817, USA; jdmcpherson@health.ucdavis.edu; 4 Comprehensive Cancer Center, University of California, Davis, Sacramento, CA 95817, USA; hogli@health.ucdavis.edu (H.L.); mbparikh@health.ucdavis.edu (M.P.); 5 Department of Public Health Sciences, University of California, Davis, Sacramento, CA 95817, USA; 6 Department of Internal Medicine, University of California, Davis, Sacramento, CA 95817, USA; 7 VA Northern California Health Care System, Sacramento, CA 95655, USA

**Keywords:** prostate cancer, PARP inhibitor, AR-pathway inhibitor, ATM inhibitor, lartesertib

## Abstract

**Background/Objectives**: Combinations of PARP inhibitors (PARPi) and androgen receptor pathway inhibitors (ARPi) have led to clinical success in treating advanced prostate cancer. However, it is unclear where in the clinical paradigm these combinations will fare best, and their mechanism of action remains unclear. We sought to address open questions and explore alternative strategies to enhance PARPi efficacy. **Methods**: Viability and morphology were assessed in response to (1) abiraterone, olaparib, or combination and (2) enzalutamide, talazoparib, or combination in castration-resistant C4-2B cells and abiraterone- or enzalutamide-resistant derivative cell models (ARPi-resistant). The efficacy of the ATM inhibitor lartesertib with and without a PARPi was also determined. Western blots and RNA-sequencing were used to interrogate the mechanistic effects of treatment. **Results**: PARPi and ARPi combinations were effective in all models but provided the most benefit in ARPi-sensitive C4-2B cells. Mechanistically, ARPi was not found to affect homologous recombination repair gene expression but may increase PARP activity. Prolonged PARP inhibition was found to increase the expression of AR target genes, and PARPi pre-treatment increased sensitivity to enzalutamide. ATM inhibition significantly increases PARPi efficacy and appears to outperform ARPi-containing combinations in ARPi-resistant models. **Conclusions**: PARPi and ARPi combinations are effective in ARPi-resistant models, but efficacy appears stronger in ARPi-sensitive CRPC cells. Presented findings support a novel hypothesis that PARP inhibition may increase ARPi sensitivity with increasing AR activity. Additionally, ATM inhibition may provide more benefit than an ARPi in combination with a PARPi in ARPi-resistant settings. These findings support continued PARPi development for improving patient outcomes.

## 1. Introduction

Poly (ADP-ribose) polymerase (PARP) inhibition has improved the treatment of a subset of patients with advanced prostate tumors [1]. Still, the PARP inhibitor (PARPi = PARP inhibitor(s)) mechanism of action (MOA) remains incompletely understood. It is generally thought that PARPi induces replication stress and DNA double-strand break accumulation [2,3,4,5]. In tumor cells deficient in homologous recombination repair (HRR), these lesions may be inappropriately handled, leading to synthetic lethality. Based on results from the PROfound and TRITON-2 clinical trials, olaparib and rucaparib were approved for castration-resistant prostate cancer (CRPC) in 2020, respectively, for patients harboring HRR gene defects [6]. More recently, preclinical and clinical evidence have demonstrated improved efficacy when combining PARPi with androgen receptor (AR) pathway inhibitors (ARPi = AR-pathway inhibitor(s)). Large randomized clinical trials in patients with CRPC have demonstrated significant clinical benefit from combining ARPi with PARPi, leading to the FDA approval of abiraterone + olaparib, enzalutamide + talazoparib, and abiraterone + niraparib [7,8,9].

Despite clinical success, questions remain regarding the optimal use of PARPi and ARPi combinations. It is unclear where in the clinical paradigm these combinations will be most effective. At the time of trials which led to the approval of PARPi and ARPi combinations, recruited CRPC patients were largely ARPi-naïve [1]. However, the clinical paradigm has changed based on trial results showing the benefit of intensifying traditional ADT with ARPi in the castration-sensitive setting [10]. Thus, many patients will have seen an ARPi prior to castration resistance. Whether combining a PARPi with an ARPi will add benefit for patients progressing on an ARPi is unclear. Clinical efforts to assess PARPi combinations in earlier, castration-sensitive settings are ongoing. The AMPLITUDE Phase 3 trial demonstrated significant benefit with the addition of niraparib to androgen deprivation therapy and abiraterone + prednisone in patients with castration-sensitive prostate cancer (CSPC) and HRR alterations, leading to the FDA approval of this combination [11].

The mechanism underlying combination efficacy remains poorly understood, but hypotheses have been postulated to explain observed effects [12]. It is thought that AR regulates DNA repair genes, including some that are involved in HRR [13]. Interestingly, previous reports showed that AR-pathway inhibition reduces HRR gene expression [14,15]. This is expected to create “BRCAness” or HRR deficiency, which increases PARPi sensitivity. In contrast to these findings, a recent study did not find evidence for reduced expression of DNA repair genes in response to AR-pathway inhibition [16]. AR-pathway inhibition was shown to increase PARP activation, presumably as compensation for decreased HRR capacity [15]. It is possible that this would increase PARPi sensitivity. PARP inhibition was shown to regulate AR transcriptional activity. Schiewer et al. demonstrated that PARP inhibition reduced the expression of AR target genes [17]. They propose a mechanistic model in which PARP inhibition reduces PARP-1-dependent GATA2 binding, which reduces AR association with chromatin. Gui et al. reported that specific inhibition of PARP-2 reduces FOXA1-dependent AR transcriptional output [18]. Thus, PARP inhibition may reduce pioneer factor function, leading to reduced AR activity. Still, how these drug classes elicit patient benefit in combination is unclear. A better understanding of cellular responses to single-agent and combination treatments will increase clinical utility. In this study, we sought to utilize ARPi-sensitive and -resistant models to address open questions in the field.

## 2. Materials and Methods

### 2.1. Cell Culture, Reagents, and Drugs

C4-2B cells were provided by Dr. Allen Gao (UC Davis) and authenticated via ATCC STR profiling service. AbiR and MDVR abiraterone- and enzalutamide-resistant C4-2B derivatives were previously described [19,20]. Briefly, C4-2B cells were chronically exposed to increasing doses of either abiraterone or enzalutamide over the course of ~12 months. LNCaP (CRL-1740-LUC2) cells were obtained from ATCC (Manassas, VA, USA). All cell lines are routinely tested for mycoplasma using InvivoGen MycoStrip tests (InvivoGen, San Diego, CA, USA, Cat#: rep-mys-20). All experiments with these cell lines and their derivatives were conducted within 15 passages after resuscitation from cryopreservation. Cells are maintained in RPMI 1640 media (Corning, Manassas, VA, USA, Cat#: 10040CV) supplemented with 10% fetal bovine serum (Corning, collected in Mexico, processed in Woodland, CA, USA, Cat#: 35010CV) and 1% penicillin/streptomycin (Gibco, Grand Island, NY, USA, Cat#: 15140122) and kept at 37 °C in a humidified incubator with 5% carbon dioxide. Olaparib (Cat#: S1060) was purchased from Selleckchem (Houston, TX, USA). Talazoparib (Cat#: HY-16106), abiraterone acetate (Cat#: HY-75054), enzalutamide (Cat#: HY-70002), and lartesertib (Cat#: HY-150617) were purchased from MedChemExpress (Monmouth Junction, NJ, USA). Cells were imaged using an Echo Revolve (Discover Echo, San Diego, CA, USA) microscope in an inverted mode at 10× magnification with 100% zoom. Images were processed and analyzed using ImageJ (version 1.54r, National Institutes of Health, Bethesda, MD, USA) [21].

### 2.2. Cell Viability Assays

Cells were plated at 5000–15,000 cells/well in 24-well plates in complete medium without a selection agent 24 h before drug administration. Cell viability was assessed 120 h after treatment using Cell Counting Kit-8 (CCK-8) (Dojindo, Kumamoto, Japan, Cat#: CK04-20). At the endpoint, the culture medium was replaced with RPMI 1640 (Gibco, Grand Island, NY, USA, Cat#:11835030) containing a 1:20 dilution of CCK-8 solution. Cells were incubated for ~1 h at 37 °C. After incubation, the medium was transferred to a 96-well plate, and the absorbance was measured at 450 nm using a SpectraMax iD5 microplate reader (Molecular Devices, San Jose, CA, USA). All conditions were performed in quadruplicate. Data is displayed as % of control cell growth +/− standard deviation. The data presented is representative of 3 independent experiments.

### 2.3. Western Blotting

Whole cell lysates were prepared by lysing with RIPA buffer (Genesee Scientific, El Cajon, CA, USA, Cat#:18-415) supplemented with 1 mM EGTA (ThermoScientific, Waltham, MA, USA, Cat#: 560767.AD), 1X Phosphatase Inhibitor Cocktail II (MedChemExpress, Monmouth Junction, NJ, USA, Cat#: HY-K0022), and 1X Halt Protease Inhibitor Cocktail with 5 mM EDTA (ThermoFisher, Waltham, MA, USA, Cat#: 78429). Coomassie (Bradford) Protein Assay Kit (ThermoFisher, Rockford, IL, USA, Cat#: 23200) was used to obtain protein concentrations. Proteins were resolved utilizing SDS-PAGE and the following primary antibodies purchased from Cell Signaling (Danvers, MA, USA) were used for detection; PAR (Cat#: 83732), PSA (Cat#: 2475), cleaved-PARP (Cat#: 9541), p21 (Cat#: 2947), γH2AX (Cat#: 9718), BRCA1 (Cat#: 14823), BRCA2 (Cat#: 10741), ATM (Cat#: 2873), phospho-ATM Ser1981 (Cat#: 13050), p53 (Cat#: 9282), phospho-p53 Ser15 (Cat#: 9284), and β-Tubulin (Cat#: 2128). Loading was checked post-transfer via Ponceau S staining, and Tubulin served as an internal loading control. Proteins were detected using Immobilon Western HRP Chemiluminescence Substrate (MilliporeSigma, Burlington, MA, USA, Cat#: WBLUF0500). All blots are representative of three independent experiments.

### 2.4. RNA-Sequencing Sample Preparation and RNA Isolation

Cells were plated and treated 24 h after plating. Five days post-treatment, RNA was isolated from cultured cells using Trizol reagent (Invitrogen, Waltham, MA, USA, Cat#: 15596018) following the manufacturer’s instructions and further purified using the RNeasy kit (Qiagen, Germantown, MD, USA, Cat#: 74104) with the optional on-column DNase step according to the manufacturer’s protocols. Total RNA was eluted from the columns in nuclease-free water. RNA concentration and purity were assessed via NanoDrop 2000 Spectrophotometer (Thermo Scientific), and quality assessments (e.g., RNA integrity) were made using an Agilent 2100 Bioanalyzer (Agilent Technologies, Santa Clara, CA, USA).

### 2.5. RNA Sequencing (RNA-Seq)

Indexed, stranded mRNA-seq libraries were prepared from total RNA (1000 ng) using the KAPA Stranded mRNA-Seq Kit (Roche, Wilmington, MA, USA) according to the manufacturer’s standard protocol for mRNA capture, fragmentation, random-primed first-strand synthesis, second-strand synthesis with dUTP marking, A-tailing, adaptor ligation, and library amplification. Libraries were pooled and multiplex sequenced on an Illumina NovaSeqX Plus System (150-bp, paired-end, >30 × 10^6^ reads per sample).

### 2.6. RNA-Seq Analysis and Bioinformatics

Raw sequencing data were processed using the nf-core/rnaseq pipeline (version 3.14.0, DOI 10.5281/zenodo.10471647), which includes quality control, alignment, and quantification steps [22]. Paired-end reads were trimmed using Trim Galore! (v0.6.7, DOI 10.5281/zenodo.5127899), a wrapper for Cutadapt (DOI:10.14806/ej.17.1.200) and FastQC (v0.12.0), which automatically detected and removed adapter sequences and trimmed low-quality bases from the 3′ ends of reads using a Phred quality score cutoff of 20. Read pairs in which either mate fell below a minimum post-trimming length of 20 bp were discarded. Read quality was assessed before and after trimming using FastQC (v0.12.0), which evaluated per-base sequence quality, GC content, sequence duplication levels, and the presence of overrepresented sequences. For alignment, reads were mapped to the *Homo sapiens* reference genome (GRCh38) using STAR [23]. Gene-level quantification was performed using Salmon against the GENCODE v45 gene annotation [24]. The pipeline also included additional quality assessment with RSeQC and featureCounts for read summarization [25,26]. All software and pipelines were run using Nextflow [27]. RNA-seq data are available via the GEO database (Accession#: GSE322615) [28].

Subsequent bioinformatic analyses were conducted in the R open source software v3.6.0 (R Core Team (2024). _R: A Language and Environment for Statistical Computing. R Foundation for Statistical Computing, Vienna, Austria. <https://www.R-project.org/>, accessed on 10 January 2026). The RNA sequencing data were quality checked and post-processed using DESeq2 v1.40.2 [29]. Euclidean distances were calculated on variance-stabilized values using a parametric fit and clustered using hclust from the stats v3.6.2 package. Differential expression analysis was carried out using a local regression fit. All code used in this project is freely available on GitHub (https://github.com/marionhardy/AK_C42B_LLG037-057) (accessed on 10 January 2026).

GSEA was conducted using the desktop software (version 4.3.3) on Hallmark, and KEGG gene sets [30,31].

### 2.7. Statistics and Schematics

All statistical testing and graphing were performed using GraphPad Prism (v.10; GraphPad Software, Boston, MA, USA) unless otherwise noted. Statistical significance (*p*-values) was calculated using an unpaired two-tailed *t*-test or ordinary one-way ANOVA followed by Dunnett’s or Sidak’s multiple comparison tests, as appropriate and as indicated in the figure legends. Outliers were identified and removed using Grubbs’ test (α = 0.05) prior to analysis. All outlier exclusions are specified in the corresponding figure legends. ns = not significant, * = *p*-value ≤ 0.05, ** = *p*-value ≤ 0.01, *** = *p*-value ≤ 0.001. All illustrations were created using BioRender.

## 3. Results

### 3.1. Abiraterone and Olaparib Combination Is Still Effective Post ARPi Exposure

**Figure 1:**
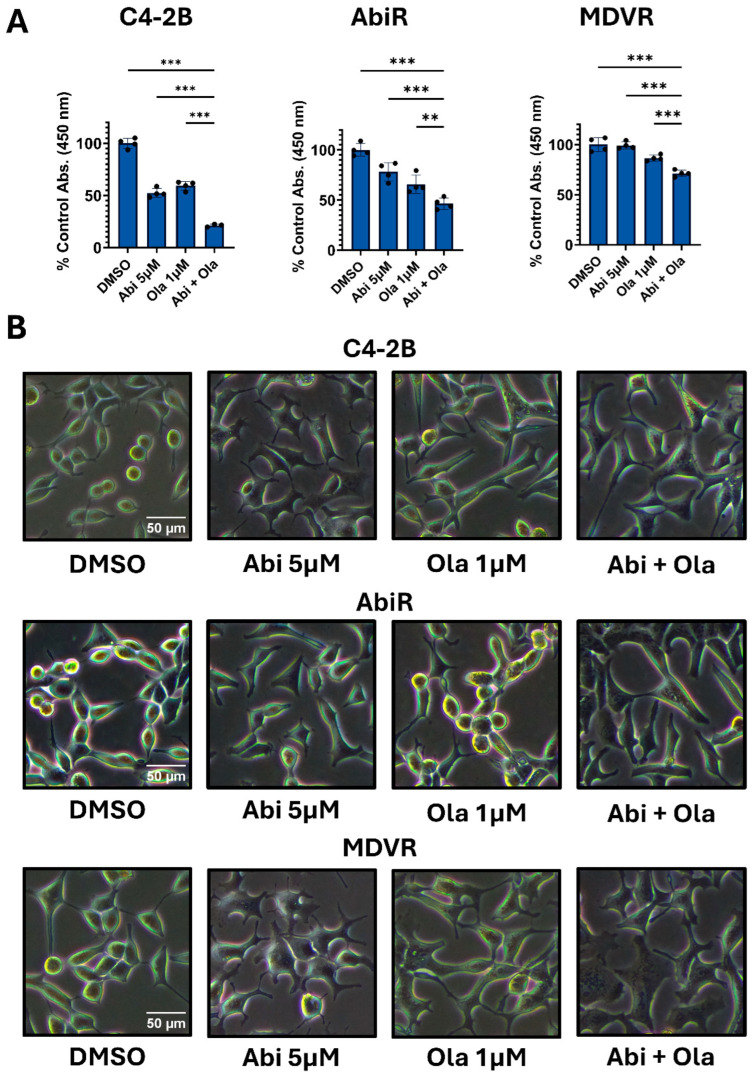
Abiraterone combined with olaparib is effective in ARPi-sensitive and -resistant prostate tumor cells. (**A**). C4-2B, AbiR, and MDVR cells were treated as indicated, and viability was determined after 5 days of continuous treatment via CCK-8. Data is presented as a % of control viability +/− standard deviation (n = 4 per condition, unless otherwise noted). Statistical analysis was performed using ordinary one-way ANOVA with Dunnett’s multiple comparisons test. Outliers were identified and removed using Grubbs’ test (α = 0.05) prior to analysis. One outlier was removed from the C4-2B Abi + Ola condition. ** = *p*-value ≤ 0.01, *** = *p*-value ≤ 0.001. Data are representative of three independent experiments. (**B**). Phase contrast microscopy was used to image the respective cell line models under the indicated treatments after 5 days. Scale bars were created using ImageJ.

Combining PARPi with ARPi is effective for the treatment of CRPC [1]. However, questions remain as the treatment paradigm for advanced prostate cancer has evolved. ARPi are often given with traditional ADT for treating castration-sensitive disease [10]. Whether an ARPi will add benefit to a PARPi post-progression on an ARPi is unclear. To address this, we treated PARPi-sensitive C4-2B cells, a CRPC model, and abiraterone- and enzalutamide-resistant C4-2B-derived cells, AbiR and MDVR, respectively, with either abiraterone (5 μM), olaparib (1 μM), or both (approved via PROpel) (Figure 1A) [19,20,32]. The combination more effectively reduced viability in all three cell lines. Observation of cell morphology supports the increased efficacy of combination treatment (Figure 1B). Of note, these cells have been shown to express CYP17A1 (abiraterone target), and previous data and those shown below support on-target in vitro effects [33,34,35]. Additionally, C4-2B was derived from LNCaP, a CSPC model shown to exhibit sensitivity to clinically relevant PARPi dosing, likely due to mutations in HRR-associated genes, including BRCA2 [36,37]. Despite the observed response in all three models, the overall effect was strongest in ARPi-sensitive C4-2B cells. These data suggest (1) that ARPi may still provide benefit in combination with a PARPi after progressing on an ARPi, but (2) combinations may be best given before use of an ARPi.

### 3.2. Characterizing Molecular Responses to Combination Treatments

**Figure 2:**
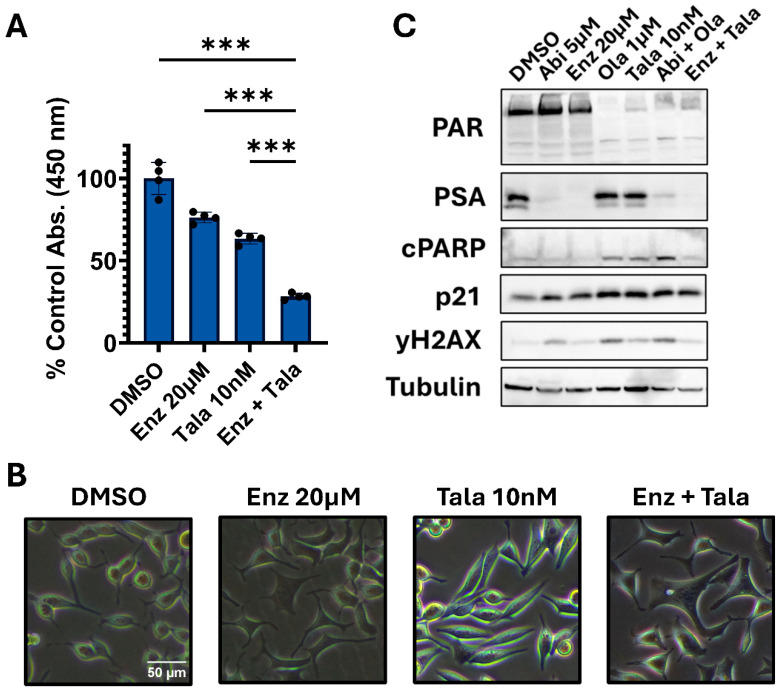
PARPi and ARPi combinations may not function as previously described. (**A**). C4-2B cells were treated as indicated, and viability was determined after 5 days of continuous treatment via CCK-8. Data is presented as a % of control viability +/− standard deviation (n = 4 per condition). Data were analyzed using ordinary one-way ANOVA with Dunnett’s multiple comparison test. No outliers were detected. *** = *p*-value ≤ 0.001. Data are representative of three independent experiments. (**B**). Phase-contrast microscopy was used to image C4-2B cells under the indicated treatments for 5 days. The scale bar was created using ImageJ. (**C**). C4-2B cells were treated as indicated for 5 days. Whole-cell lysates were prepared and assayed via Western blot to assess expression of the indicated markers. Western blots are representative of 3 independent experiments.

In line with clinical data and results presented above, combining enzalutamide and talazoparib (approved via TALAPRO-2) is also more effective than monotherapy in C4-2B cells (Figure 2A,B). It remains unclear what underlies the efficacy of combining these drug classes. One hypothesis is that ARPi reduces HRR, induces or enhances BRCAness, and sensitizes to PARP inhibition [12,14,15]. We’ve previously shown increased expression of γH2AX (indicating DNA damage), p21 (indicating cell-cycle arrest), and cleaved-PARP (indicating cell death) in response to PARP inhibition, in line with expected PARPi function [37]. If ARPi reduced HRR, we hypothesize that the combination with a PARPi would further increase expression of these markers as a result of increased DNA damage. Western blots show that olaparib and talazoparib indeed lead to increased expression of noted response markers (Figure 2C). Interestingly, the addition of abiraterone to olaparib or enzalutamide to talazoparib did not consistently lead to increased marker expression. These data do not support that AR-pathway inhibition reduces HRR to enhance synthetic lethality. Altogether, these data support the efficacy of combining PARPi and ARPi but suggest that the combination MOA requires further investigation.

### 3.3. RNA-Sequencing Provides Insight into Tumor Cell Response to PARPi and ARPi Monotherapy and Combination Treatments

**Figure 3:**
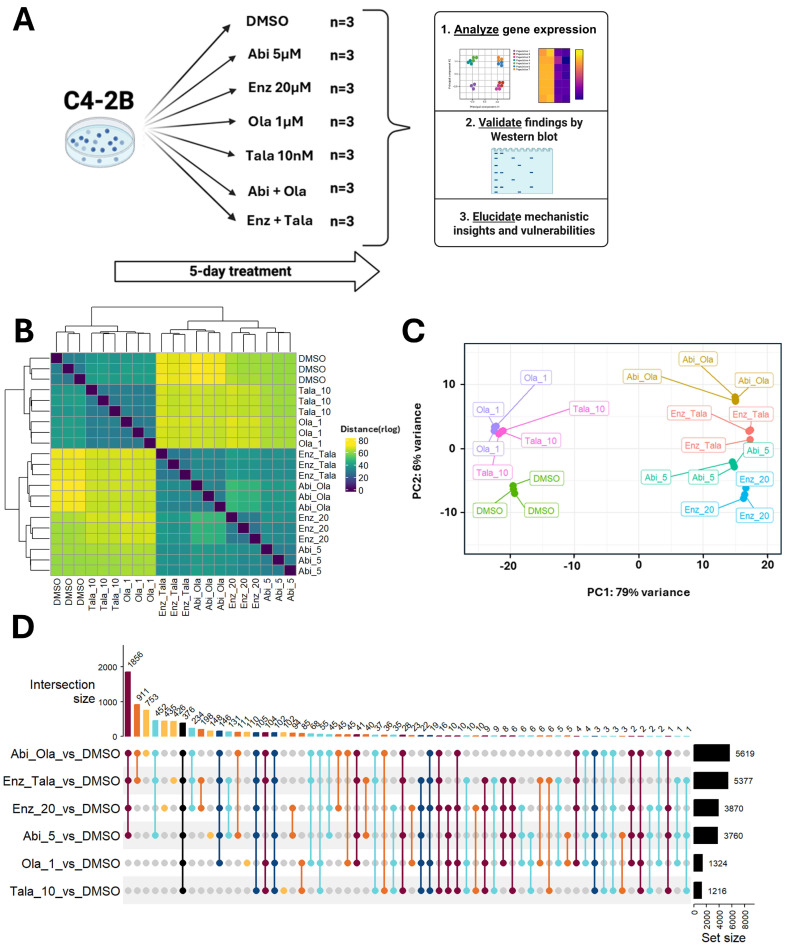
RNA-sequencing provides insight into the effects of single-agent and combination treatments. (**A**). Schematic detailing RNA-sequencing and experimental design. C4-2B cells were treated as indicated for 5 days, followed by RNA extraction, RNA-sequencing, and analysis. Created in BioRender. Hardy, M. (2026) https://BioRender.com/vuud55i (accessed 16 March 2026). (**B**). Euclidean distance plotting and (**C**). Principal component analysis (PCA) revealed treatment group transcriptomic differences. (**D**). An upset plot displays treatment group-specific and shared differentially expressed genes (DEG). The set size is defined as the total number of DEGs for a given treatment. Intersection size is the total number of unique DEGs in a given set of treatments, shown by different colored dots that connect treatment groups.

To better address combination MOA, we performed RNA-sequencing to define transcriptional responses to abiraterone, enzalutamide, olaparib, talazoparib, and respective combinations in C4-2B cells (5-day treatment) (Figure 3A). Graphing Euclidean distances demonstrates the similarity of responses to drugs of the same class; olaparib clusters with talazoparib, abiraterone with enzalutamide, and combinations (Figure 3B). Interestingly, despite having relatively similar effects on viability at chosen doses, ARPi monotherapies appear to drive much more transcriptional change than single-agent PARP inhibition. Principal component analysis further supports this observation, as ARPi largely segregates along PC1 (79% variance), while PARPi segregates along PC2 (6% variance) (Figure 3C). An upset plot, which captures the number of differentially expressed genes (DEGs) by treatment and both treatment-specific and intersection-specific DEG changes, shows that AR-pathway inhibition results in greater numbers of DEGs versus PARPi, while combinations exhibit the most overall DEGs (Figure 3D).

To gain further insight into the effects of treatments, we performed gene set enrichment analysis (GSEA) and focused on interrogation of significantly enriched hallmark and KEGG gene sets either up- or downregulated common to each treatment class: ARPi = abiraterone and enzalutamide, PARPi = olaparib and talazoparib, combination = abiraterone + olaparib and enzalutamide + talazoparib (Supplementary Materials Figures S1 and S2). Both ARPi and PARPi commonly up- or downregulate several hallmark gene signatures (e.g., upregulated: interferon-α/γ, apoptosis, epithelial-mesenchymal transition, downregulated: Myc, E2F targets, unfolded protein response), which may imply a common stress response. As expected, ARPi significantly reduced androgen response gene expression, while PARPi strongly induced the expression of p53-regulated genes. Notable differences include increased Wnt signaling associated with ARPi-treated cells and more downregulation of pathways directly associated with cell cycle regulation in PARPi-treated cells. These data are also reflected in KEGG gene set enrichment patterns. We also observe striking metabolic differences, where ARPi more generally downregulate, and PARPi more generally upregulate varying metabolism-related gene expression. Interestingly, while ARPi downregulates androgen response signaling as expected, PARPi increases this expression. Combinations are amalgamations of both monotherapies, but appear to favor the expression profile exhibited by ARPi. However, combinations also exhibit notable specific alterations, including changes associated with immune cell activity and extracellular matrix interactions. Altogether, these findings support known drug function and provide a foundation to further probe the specific mechanisms underlying the efficacy of combinations.

### 3.4. ARPi Do Not Downregulate HRR Associated Gene Expression but May Increase PARP Activity

**Figure 4:**
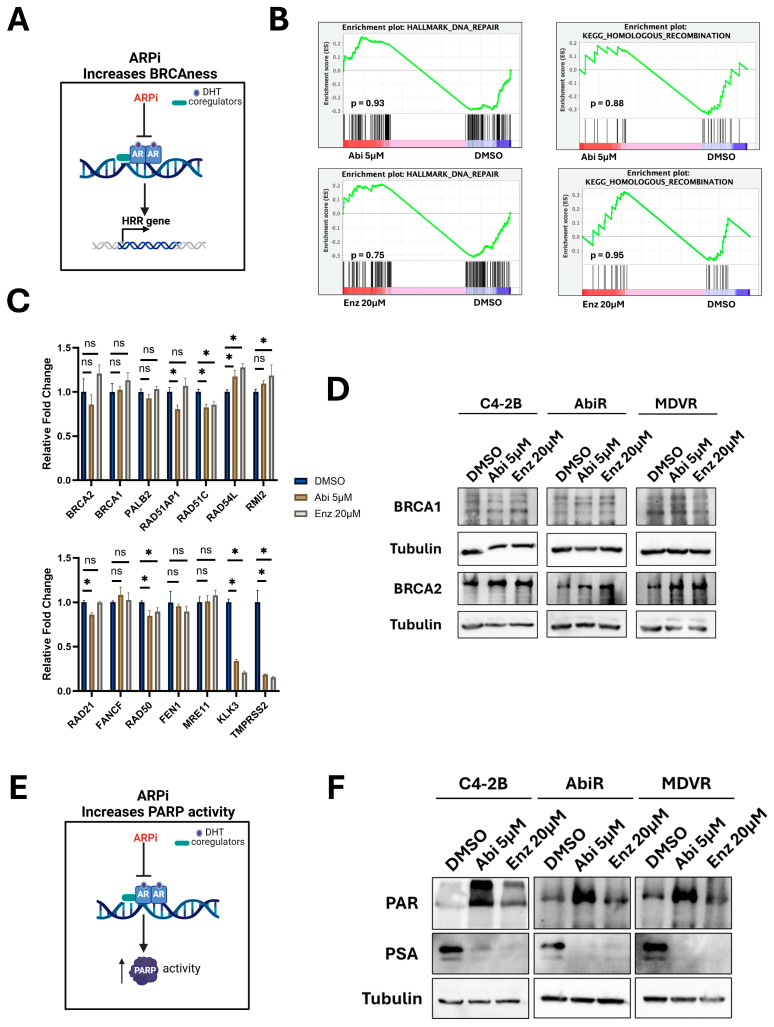
ARPi does not consistently down-regulate HRR genes but may positively induce PARP activity. (**A**). Schematic representation of a hypothesis stating that AR-pathway inhibition may down-regulate HRR gene expression, leading to “BRCAness.” Created in BioRender. Hardy, M. (2026) https://BioRender.com/vuud55i (accessed 16 March 2026). (**B**). GSEA interrogated hallmark DNA repair or KEGG homologous recombination gene sets in response to either abiraterone or enzalutamide. (**C**). Analysis of RNA-seq read counts (averages of groups normalized to DMSO) for the indicated HRR genes. KLK3 and TMPRSS2 serve as positive controls for abiraterone and enzalutamide treatment. Data were analyzed using ordinary one-way ANOVA with Dunnett’s multiple comparison test (n = 3). * = *p*-value ≤ 0.05, ns = not significant. (**D**). Western blots reveal the expression of BRCA1 or BRCA2 in response to abiraterone or enzalutamide in the indicated cell line models after 5 days of treatment. Tubulin served as a loading control. Western blots are representative of 3 independent experiments. (**E**). Schematic representation of a hypothesis stating that ARPi may induce PARP activity, which could increase PARPi sensitivity. Created in BioRender. Hardy, M. (2026) https://BioRender.com/vuud55i (accessed 16 March 2026). (**F**). Western blots reveal PAR levels and PSA expression in response to abiraterone or enzalutamide in the indicated cell line models after 5 days of treatment. Tubulin served as a loading control. Western blots are representative of 3 independent experiments.

Several hypotheses have been proposed to explain the efficacy of combining PARPi and ARPi [12]. AR is thought to regulate HRR, and AR-pathway inhibition has been shown to down-regulate HRR-associated gene expression [14,15]. Thus, a leading hypothesis is that ARPi decreases HRR, induces BRCAness, and sensitizes to PARP inhibition (Figure 4A). To address this hypothesis, we interrogated our RNA-seq data for evidence that ARPi decreases HRR gene expression. We found no significant downregulation of either the hallmark DNA repair or KEGG homologous recombination gene sets in response to either abiraterone or enzalutamide (Figure 4B). We analyzed the expression of a set of genes previously highlighted to be downregulated by inhibition of AR activity and additionally looked at BRCA2 and PALB2, two genes associated with PARPi sensitivity [14,15,38]. Neither abiraterone nor enzalutamide consistently or robustly downregulated their expression, while positive control genes (KLK3 (PSA), TMPRSS2) were strongly repressed by both drugs (Figure 4C). Lastly, we specifically tested for the expression of both BRCA1 and BRCA2 in response to ARPi treatment at the protein level (Figure 4D). In line with transcriptomic data, we saw no significant evidence that ARPi downregulates their expression in C4-2B or ARPi-resistant cells. Collectively, these data do not support that ARPi induces PARPi sensitivity through downregulation of HRR.

It has been shown that AR-pathway inhibition increases PARP activity (Figure 4E). ARPi treatment of C4-2 cells resulted in increased PAR levels, and clinical castration significantly increased the PAR/PARP1 ratio, all supporting that AR-pathway inhibition augments PARP activity [15]. To address this hypothesis, we treated C4-2B and ARPi-resistant cells with vehicle or either abiraterone (5 μM) or enzalutamide (20 μM) and assessed PAR levels (Figure 4F). Interestingly, we observed that both ARPi increased PAR levels in C4-2B cells. The results were less consistent in AbiR and MDVR cells. These data suggest that AR-pathway inhibition may increase PARP activity, which could explain some of the efficacy of combining these drugs.

### 3.5. Prolonged Exposure to PARP Inhibition Increases Androgen Response Gene Expression

**Figure 5:**
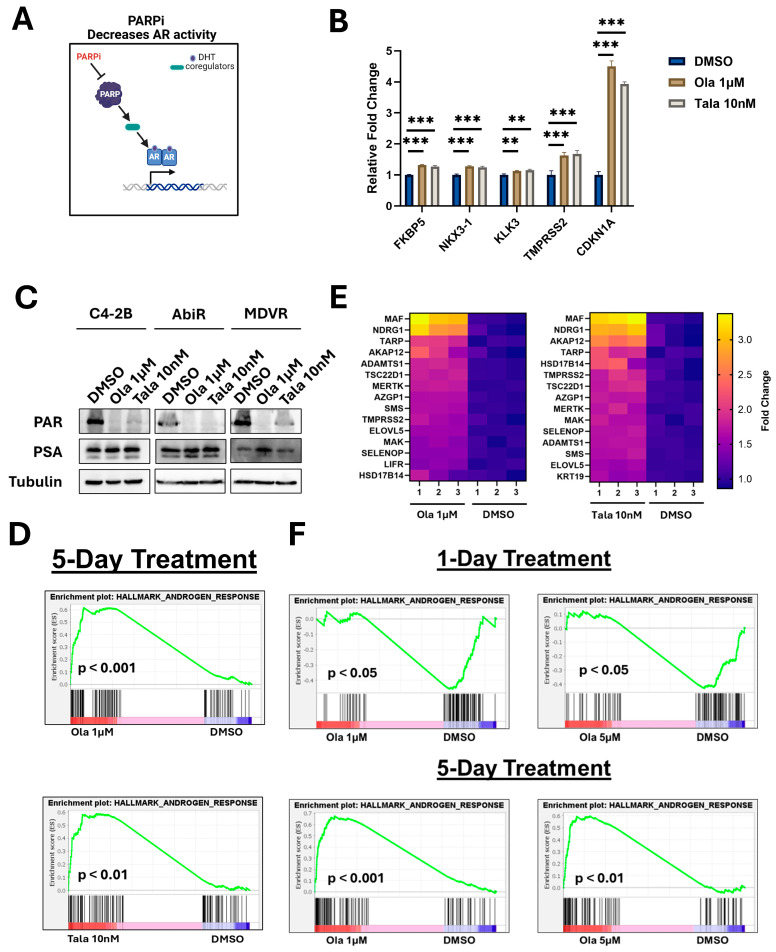
Prolonged exposure to PARP inhibition enhances AR-regulated gene expression. (**A**). Schematic representation of a hypothesis stating that PARP inhibition may reduce AR transcriptional output through regulation of AR co-regulators. Created in BioRender. Hardy, M. (2026) https://BioRender.com/vuud55i (accessed 16 March 2026). (**B**). Analysis of RNA-seq read counts (averages of groups normalized to DMSO) for the indicated AR-target genes. CDKN1A (p21) served as a positive control for olaparib and talazoparib. Data were analyzed using ordinary one-way ANOVA with Dunnett’s multiple comparison test (n = 3). ** = *p*-value ≤ 0.01, *** = *p*-value ≤ 0.001. (**C**). Western blots reveal PAR levels and PSA expression in response to olaparib or talazoparib in the indicated cell line models after 5 days of treatment. Tubulin served as a loading control. Western blots are representative of 3 independent experiments. (**D**). GSEA interrogated the hallmark androgen response gene set in response to either olaparib or talazoparib. (**E**). Heatmaps display GSEA hallmark androgen response genes at the leading edge in response to olaparib or talazoparib, indicating genes most upregulated by treatment. (**F**). GSEA interrogated the hallmark androgen response gene set in response to either 1 μM or 5 μM olaparib for either 1 or 5 days in C4-2B cells.

It is also thought that PARP positively regulates AR activity (Figure 5A). Two previous reports have presented data supporting the role of PARP1 and PARP2 in promoting AR transcriptional output [17,18]. The inhibition of PARP1 or PARP2 is thought to decrease AR transcriptional activity and thus could synergize with AR-pathway inhibition. To address this hypothesis, we first analyzed RNA-seq gene expression levels of canonical AR target genes and CDKN1A (p21, PARPi positive control) in C4-2B cells treated with olaparib (1 μM) or talazoparib (10 nM) for 5 days (Figure 5B). Interestingly, we found significantly increased expression of every gene analyzed (although the magnitude of change was modest for all AR-target genes). Western blots confirmed that neither olaparib nor talazoparib resulted in decreased expression of PSA in C4-2B or ARPi-resistant cells after 5 days of treatment (Figure 5C). GSEA revealed that PARPi treatment resulted in a significant increase in the expression of the hallmark androgen response gene set (Figure 5D). Those genes at the leading edge of the set in response to PARPi treatment are displayed (genes most upregulated by PARP inhibition) (Figure 5E). Interestingly, while there is a high concordance between these genes in response to either PARPi, there are notable similarities and differences between those most upregulated by PARP inhibition and those most downregulated by AR-pathway inhibition, which may imply a degree of AR-reprogramming in response to PARP inhibition (Supplementary Materials Figure S3A). Altogether, these data suggest that rather than decreasing AR-activity, prolonged exposure to PARP inhibition leads to increased AR transcriptional output. This increase in activity may sensitize to AR-pathway inhibition. To test this hypothesis, we pre-treated C4-2B cells for 9 days with olaparib and compared the response between parental and olaparib pre-treated cells to enzalutamide (Supplementary Materials Figure S3B). Olaparib pre-treated C4-2B cells were significantly more sensitive to enzalutamide. These data suggest there may be synergy between these agents induced by the effects of PARP inhibitor exposure.

We sought to reconcile our study with previously published data. A notable difference in our study is the treatment duration. Schiewer et al. performed many experiments either at 30 min or 24 h [17]. In contrast, we assessed at 5 days. Additionally, Schiewer et al. primarily used veliparib for their study, which we now know is not as potent a PARPi as olaparib or talazoparib [4]. To test the impact of time and treatment intensity on AR target gene expression, we performed an independent experiment by treating C4-2B cells with olaparib (1 or 5 μM) for either 1 or 5 days and performed RNA-seq (Figure 5F). After 1 day of olaparib exposure, we found significantly decreased expression of the hallmark androgen response gene set at both doses, in line with previous findings. However, by day 5, this effect was robustly reversed. Looking at specific gene expression suggests there may additionally be a dependency on dosing in addition to time (Supplementary Materials Figure S4). These data suggest that while short-term PARP inhibition may decrease AR activity, long-term exposure, and possibly adaptation, promotes AR activity. This may indicate enhanced reliance on AR signaling and sensitivity to AR-pathway inhibition. It is possible that PARPi effects on cell cycle dynamics may alter AR-target gene expression, but we have found that 1 µM olaparib induces modest changes to cell cycle distribution in C4-2B cells. This novel hypothesis merits further investigation.

### 3.6. Co-Targeting ATM Rather than AR May Be a Superior Strategy in Combination with a PARPi in ARPi-Resistant Cells

**Figure 6:**
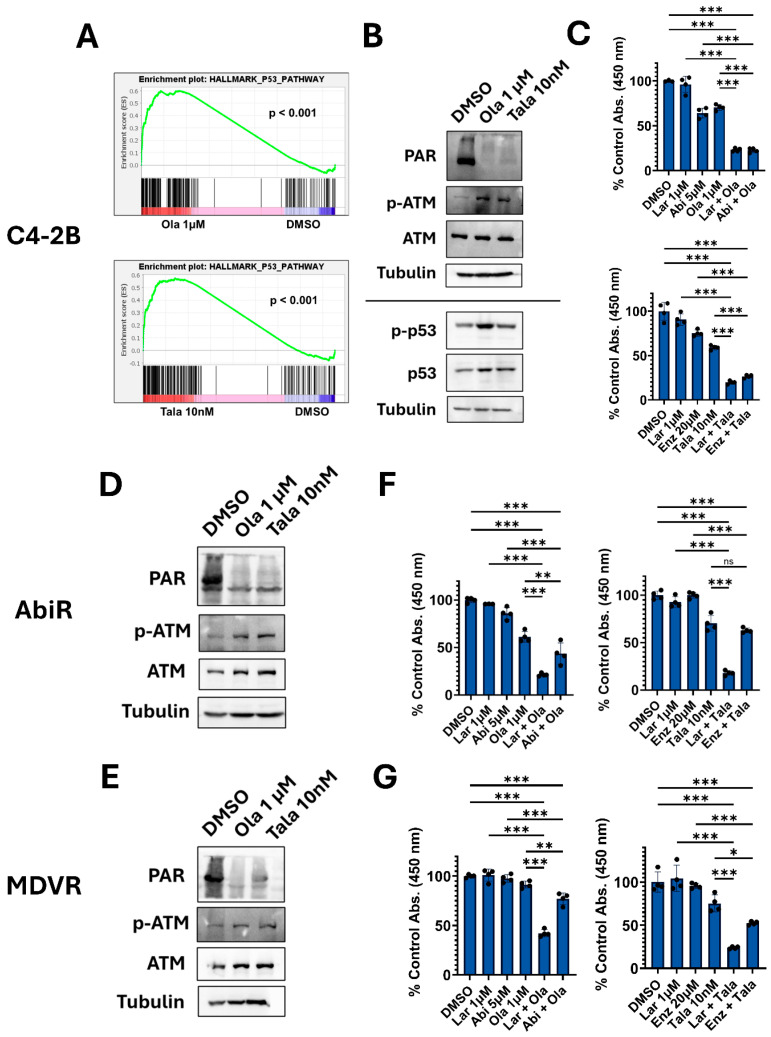
ATM inhibition significantly reduces prostate tumor cell viability in combination with a PARPi. (**A**). GSEA interrogated the hallmark p53 pathway gene set in response to either olaparib or talazoparib. (**B**). Western blots reveal the expression of indicated proteins in response to olaparib or talazoparib in C4-2B cells after 5 days of treatment. Tubulin served as a loading control. (**C**). Viability assays tested the response to indicated 5-day treatments in C4-2B cells via CCK-8. Data are presented as a % of control viability +/− standard deviation. (n = 4 per condition, unless otherwise noted). (**D**,**E**). Western blots reveal the expression of indicated proteins in response to olaparib or talazoparib in indicated cell line models after 5 days of treatment. Tubulin served as a loading control. (**F**,**G**). Viability assays tested the response to indicated 5-day treatments in indicated cell lines via CCK-8. Data is presented as a % of control viability +/− standard deviation (n = 4 per condition, unless otherwise noted). Data for panels (**C**,**F**,**G**) were analyzed using ordinary one-way ANOVA with Sidak’s multiple comparison test. Outliers were identified and removed using Grubbs’ test (α = 0.05) prior to analysis. Outliers were removed for the C4-2B panel C DMSO treatment (top graph) and AbiR panel F Lar 1 µM treatment (left graph). * = *p*-value ≤ 0.05, ** = *p*-value ≤ 0.01, *** = *p*-value ≤ 0.001, ns = not significant. Cell viability assays and western blots are all representative of three independent experiments.

While the mechanism underlying PARPi and ARPi combination efficacy remains unclear, we hypothesize that improved understanding of PARPi function may lead to the design of superior strategies. Previous data showed PARP inhibition increases yH2AX expression, indicative of DNA damage induction (Figure 2C) [37]. GSEA shows that both olaparib and talazoparib significantly increase p53 pathway expression in line with DNA damage response (DDR) activation (Figure 6A). Western blots for phosphorylation of p53 at Ser15 support GSEA data and activation of p53 signaling (Figure 6B). The DDR is regulated upstream by three members of the PI3K-related kinase family, namely, ATM, ATR, and DNA-PK [39]. Previous reports have shown that ATM may be activated by PARP inhibition and is capable of phosphorylating p53 at Ser15 [40,41,42]. Both olaparib and talazoparib treatment resulted in increased phosphorylation of ATM at Ser1981, suggesting activation (Figure 6B). We hypothesized that inhibition of ATM would impair cellular response to PARP inhibition and enhance PARPi anti-tumor effects. Combining the clinical-stage ATM inhibitor (ATMi) lartesertib with either olaparib or talazoparib significantly reduced C4-2B viability (Figure 6C). We also found that PARP inhibition resulted in increased ATM phosphorylation in ARPi-resistant cells (Figure 6D,E). Combination of lartesertib with either olaparib or talazoparib dramatically reduced the viability of both cell lines (Figure 6F,G). Excitingly, the combination of a PARPi with an ATMi outcompeted the ARPi-containing regimens in both ARPi-resistant cell lines. This suggests that in ARPi-resistant settings, an ATM inhibitor may be a superior choice in combination with a PARPi. Collectively, these data support additional studies to further define PARPi MOA and ATMi combinations.

## 4. Discussion

Combining PARPi and ARPi has demonstrated clinical efficacy in advanced prostate cancer, and ongoing clinical development seeks to provide benefit to more patients [1]. Still, important questions remain, as it is unclear where in the clinical paradigm these regimens will fare best and how these drug classes work in combination. Trials of patients with CRPC treated with ARPi and PARPi largely did not include patients previously treated with an ARPi. Whether combinations of PARPi with ARPi would provide benefit in the CRPC space, given the changed treatment paradigm of using ARPi in the castration-sensitive setting, is unclear. The presented data suggest that combination treatment may still be beneficial in patients who progress on an ARPi. A recent retrospective trial reported that patients progressing on abiraterone with DDR gene mutations had significantly longer progression-free and overall survival and higher PSA responses on a combination of abiraterone with olaparib versus olaparib alone [43]. Though the authors concede study limitations (retrospective nature, small sample size, and short follow-up) and the need for larger prospective trial confirmation, they note the findings imply synergy between these drug classes.

Whether synergy exists between PARPi and ARPi remains unclear. PROpel and TALAPRO-2 demonstrated benefit from combination treatment in biomarker-selected and unselected patients, supporting synergy [7,8]. However, MAGNITUDE did not show a benefit of niraparib with abiraterone in unselected patients, complicating the outlook [9]. Preclinical studies provide mixed results. Li et al. presented findings supporting synergy between enzalutamide and olaparib, while Asim et al. showed an apparent synergistic effect of bicalutamide and olaparib in the C4-2 xenograft model [14,15]. More recently, Illuzzi et al. showed generally higher synergy scores between enzalutamide and olaparib across various prostate cancer cell lines exhibiting moderate to high ARPi sensitivity [44]. In ARPi-resistant models, synergy scores were relatively diminished. These data support the currently presented findings, which suggest that underlying ARPi sensitivity is a factor in determining combination efficacy. Interestingly, Illuzzi et al. provide evidence to suggest that increasing PARPi sensitivity through ATM knockout also enhances combination effectiveness [44]. This is particularly interesting, as while trials have found biomarker-unselected patients may respond to combination treatment, stronger effects are seen in biomarker-positive cohorts [1]. Together, these data suggest that the efficacy of combining these drug classes may largely be driven by underlying individual sensitivity to either AR-pathway or PARP inhibition.

In contrast to the above studies, a recent report by Traphagen et al. showed that inhibition of AR not only fails to synergize with PARPi but may actually impair response to PARP inhibition in castration-sensitive LNCaP cells [16]. This study further suggests that reduced proliferation decreases PARPi efficacy. Thus, in tumor cells highly sensitive to AR-pathway inhibition, cells will slow growth and reduce response to PARP inhibition. We also tested combinations in castration-sensitive LNCaP cells. Combination treatment was more effective than monotherapy, but in line with Traphagen et al., LNCaP appeared more sensitive to ARPi compared to C4-2B and ARPi-resistant models, which blunted the overall observed combination effect (Supplementary Materials Figure S5). Regardless of whether combinations are synergistic or not, clinical evidence supports their efficacy in both castration-resistant and -sensitive settings, especially in those expected to exhibit strong PARPi responses based on biomarker selection. Traphagen et al. note that benefit may simply derive from targeting independent vulnerabilities [16]. A more complete understanding of the MOA of each drug alone and in combination may elucidate potential synergies and improve clinical utility.

Both Li et al. and Asim et al. put forth the hypothesis that AR-pathway inhibition reduces HRR gene expression and induces PARPi sensitivity [14,15]. However, the effect is inconsistent across cell lines in the Li et al. study. Illuzzi et al. present data that enzalutamide does not reduce HRR capacity, as demonstrated by assaying for RAD51 foci [44]. However, they note that the enzalutamide and olaparib combination increases DNA damage. Traphagen et al. do not find that AR-pathway inhibition reduces DNA repair gene expression in either LNCaP or 22Rv1 cells [16]. Current findings support that AR-pathway inhibition does not reduce HRR gene expression. Altogether, these studies suggest that if, in fact, AR-pathway inhibition affects DNA repair, it may be context-dependent and thus is unlikely to completely explain combination efficacy.

The present findings support a role for ARPi in inducing PARP activity, in line with data presented by Asim et al. [15]. However, abiraterone consistently outperformed enzalutamide, and the overall data were more modest in ARPi-resistant settings. More research is needed to determine the role of this effect. Previous reports also suggest PARPi may reduce AR transcriptional activity and thus may work with AR-pathway inhibition. Schiewer et al. demonstrated that PARP1 regulates AR chromatin occupancy and transcriptional output, while Gui et al. highlighted a specific role for PARP2 in mediating FOXA1-dependent AR activity [17,18]. Additionally, Traphagen et al. present data that support PARP inhibition modulates AR-regulated genes [16]. In contrast, Illuzzi et al. report that olaparib does not change AR-regulated gene expression [44]. The current study shows that the effect of PARP inhibition on AR may be time-dependent, with prolonged PARPi exposure resulting in increased AR activity. It is possible that PARPi-induced AR activity may enhance ARPi-sensitivity. Indeed, pre-treatment of C4-2B cells with olaparib increased enzalutamide sensitivity, which suggests potential synergy between these agents. This merits further investigation, as it may have significant clinical implications.

Altogether, preclinical and clinical findings support PARPi and ARPi combination utility, but suggest efficacy depends on underlying sensitivity to individual drugs, especially PARPi. Despite improved outcomes in biomarker-unselected groups, responses are greater for biomarker-positive patients [1]. Even in the AMPLITUDE trial, which recently demonstrated the efficacy of niraparib combined with abiraterone in castration-sensitive patients harboring HRR gene alterations, responses were best in the BRCA subgroup, and approval was granted only for BRCA2 mutations by the FDA [11]. These findings underscore the importance of improving understanding of PARP inhibition and what underlies PARPi efficacy. While certain biomarkers appear to predispose to PARP inhibition, it remains unclear how PARPi works. The prevailing thought is that PARPi induces replication stress and DNA damage, which requires HRR, but how PARPi induces replication stress is incompletely understood [2,3,4,5,45]. Interestingly, emerging evidence suggests the concept of BRCAness may not be reliant on impaired HRR, but rather on impaired replication gap suppression [46,47]. An improved understanding of PARPi MOA promises to improve our ability to use PARPi, which will enhance combination design.

This study does support that PARP inhibition induces DNA damage, resulting in ATM activation. Co-inhibition of ATM leads to an apparent synergistic reduction in cell viability and is shown to outcompete the efficacy of ARPi-containing combinations in ARPi-resistant models. These data have important implications. First, they support that improved treatments may be created through rational co-targeting of PARPi MOA. Secondly, they support the continued development of this strategy for patients who may derive benefit from a PARPi but who may respond poorly to AR-pathway inhibition. Recent studies have shown that PARP inhibition may be enhanced in combination with ATM inhibitors, and preclinical evidence suggests the combinations are safe [48,49].

## 5. Conclusions

This study addresses open questions and supports the ongoing development of PARPi and ARPi combinations for the treatment of prostate cancer. The presented work supports clinical findings that combinations may be beneficial even after progression on an ARPi but underscores the need to further define the MOA [43]. Current data do not support the hypotheses that ARPi reduces HRR capacity or that PARPi meaningfully reduces AR activity. Rather, data support a novel hypothesis that prolonged PARPi treatment may augment AR activity and sensitivity to AR-pathway inhibition. This hypothesis may highlight a previously unknown synergy between these agents and may help explain clinical findings. These data suggest that combination efficacy may depend on underlying PARPi sensitivity rather than the ability of an ARPi to sensitize to PARP inhibition. Future work should be directed at improving the understanding of PARPi sensitivity and how this influences the benefit from combination with an ARPi. Combined with previous and current findings suggesting that PARPi efficacy depends on cell proliferation, we propose that the development of PARPi lead-in strategies prior to ARPi administration may prove to be able to maximize combination effectiveness [16]. Lastly, this study suggests that targeting ATM in combination with a PARPi may be a viable alternative strategy to increase PARPi utility. Future work is needed to explore the translation of PARPi and ATMi combinations. Understanding possible increases in toxicity will be key to understanding the potential of this treatment.

## Data Availability

The original contributions presented in this study are included in the article/Supplementary Material. Further inquiries regarding raw data supporting this study can be directed to the corresponding author, and data are available upon request.

## Supplementary Materials

The following supporting information can be downloaded at: https://www.mdpi.com/article/10.3390/biomedicines14050949/s1, Figure S1: Hallmark gene set enrichment analysis (GSEA). Hallmark gene sets were investigated by GSEA in samples by groups; ARPi = abiraterone and enzalutamide, PARPi = olaparib and talazoparib, combination = abiraterone + olaparib and enzalutamide + talazoparib. Displayed are gene sets which are both significantly (*p* < 0.05) and either commonly up- or downregulated by group. Figure S2: KEGG gene set enrichment analysis (GSEA). KEGG gene sets were investigated by GSEA in samples by groups; ARPi = abiraterone and enzalutamide, PARPi = olaparib and talazoparib, combination = abiraterone + olaparib and enzalutamide + talazoparib. Displayed are gene sets which are both significantly (*p* < 0.05) and either commonly up- or downregulated by group. Figure S3: Insights into hypothesis that PARP inhibition augments AR transcriptional activity. (A). Heatmaps display GSEA hallmark androgen response genes at the leading edge in response to abiraterone or enzalutamide indicating genes most downregulated by treatment in C4-2B cells. (B). C4-2B cells were pre-treated with olaparib (5 µM) for 9 days and subsequently re-plated and tested for enzalutamide sensitivity compared to parental C4-2B cells. Viability was determined after 5-days of enzalutamide treatment via CCK-8. Data is presented as a % of control viability +/− standard deviation (n = 4 per condition). Data was analyzed using unpaired two-tailed *t*-test. *** = *p*-value ≤ 0.001. Data is representative of three independent experiments. Figure S4: Assessing impact of olaparib treatment duration and intensity on AR-target gene expression. C4-2B cells were treated with either 1 μM or 5 μM olaparib for (A). 1 or (B). 5 days. Analysis of RNA-seq read counts (averages of groups normalized to DMSO) for indicated AR-target genes is displayed. CDKN1A (p21) served as a positive control for olaparib treatment. Data is presented as a % of control viability +/− standard deviation (n = 3). Data was analyzed using ordinary one-way ANOVA followed by Dunnett’s multiple comparison, * = *p*-value ≤ 0.05, ** = *p*-value ≤ 0.01, *** = *p*-value ≤ 0.001, ns = not significant. Figure S5: PARPi and ARPi combination efficacy testing in LNCaP cells. Viability assays tested response to indicated 5-day treatments in LNCaP cells via CCK-8. Data is presented as a % of control viability +/− standard deviation (n = 4, unless otherwise indicated). Data was analyzed using ordinary one-way ANOVA followed by Dunnett’s multiple comparison test. Outliers were identified and removed via Grubb’s test (α = 0.05). One outlier was removed from the LNCaP Enz 20 µM condition (right graph). * = *p*-value ≤ 0.05, *** = *p*-value ≤ 0.001, ns = not significant. Data is representative of three independent experiments.
