# Supplementary material for: Characterizing Response to PARP Inhibitor Treatment Combinations in Advanced Prostate Cancer
Source: Biomedicines. 2026 Apr 22;14(5):949. doi: 10.3390/biomedicines14050949 (PMC13203762; doi:10.3390/biomedicines14050949)
Supplement: Supplementary file 1 [file biomedicines-14-00949-s001.zip › biomedicines-4207950-supplementary.pdf]

HALLMARK Positively Enriched in both Abiraterone and Enzalutamide
HALLMARK_INTERFERON_ALPHA_RESPONSE
HALLMARK_INTERFERON_GAMMA_RESPONSE
HALLMARK_WNT_BETA_CATENIN_SIGNALING
HALLMARK_APOPTOSIS
HALLMARK_EPITHELIAL_MESENCHYMAL_TRANSITION
HALLMARK_KRAS_SIGNALING_DN
HALLMARK_MYOGENESIS

HALLMARK Negatively Enriched in both Abiraterone and Enzalutamide
HALLMARK_ANDROGEN_RESPONSE
HALLMARK_MYC_TARGETS_V1
HALLMARK_MYC_TARGETS_V2
HALLMARK_OXIDATIVE_PHOSPHORYLATION
HALLMARK_MTORC1_SIGNALING
HALLMARK_UNFOLDED_PROTEIN_RESPONSE
HALLMARK_E2F_TARGETS

HALLMARK Positively Enriched in both Olaparib and Talazoparib
HALLMARK_P53_PATHWAY
HALLMARK_ANDROGEN_RESPONSE
HALLMARK_EPITHELIAL_MESENCHYMAL_TRANSITION
HALLMARK_HYPOXIA
HALLMARK_TNFA_SIGNALING_VIA_NFKB
HALLMARK_FATTY_ACID_METABOLISM
HALLMARK_INTERFERON_ALPHA_RESPONSE
HALLMARK_INTERFERON_GAMMA_RESPONSE
HALLMARK_APOPTOSIS
HALLMARK_MYOGENESIS
HALLMARK_BILE_ACID_METABOLISM
HALLMARK_COAGULATION

HALLMARK Negatively Enriched in both Olaparib and Talazoparib
HALLMARK_E2F_TARGETS
HALLMARK_G2M_CHECKPOINT
HALLMARK_MYC_TARGETS_V1
HALLMARK_UNFOLDED_PROTEIN_RESPONSE
HALLMARK_MITOTIC_SPINDLE

HALLMARK Positively Enriched in both Abi+Ola and Enz+Tala
HALLMARK_INTERFERON_ALPHA_RESPONSE
HALLMARK_INTERFERON_GAMMA_RESPONSE
HALLMARK_P53_PATHWAY
HALLMARK_APOPTOSIS
HALLMARK_EPITHELIAL_MESENCHYMAL_TRANSITION
HALLMARK_KRAS_SIGNALING_DN
HALLMARK_MYOGENESIS
HALLMARK_APICAL_JUNCTION

HALLMARK Negatively Enriched in both Abi+Ola and Enz+Tala
HALLMARK_ANDROGEN_RESPONSE
HALLMARK_MYC_TARGETS_V1
HALLMARK_MYC_TARGETS_V2
HALLMARK_E2F_TARGETS
HALLMARK_G2M_CHECKPOINT
HALLMARK_UNFOLDED_PROTEIN_RESPONSE
HALLMARK_MTORC1_SIGNALING
HALLMARK_OXIDATIVE_PHOSPHORYLATION

Supplemental Figure S1: Hallmark gene set enrichment analysis (GSEA). Hallmark gene sets were investigated by GSEA in samples by groups; ARPi = abiraterone and enzalutamide, PARPi = olaparib and talazoparib, combination = abiraterone + olaparib and enzalutamide + talazoparib. Displayed are gene sets which are both significantly ($p < 0.05$) and either commonly up- or downregulated by group.

KEGG Positively Enriched in both Abiraterone and Enzalutamide
KEGG_TASTE_TRANSDUCTION
KEGG_AXON_GUIDANCE
KEGG_CELL_ADHESION_MOLECULES_CAMS
KEGG_WNT_SIGNALING_PATHWAY
KEGG_ERBB_SIGNALING_PATHWAY
KEGG_BASAL_CELL_CARCINOMA
KEGG_CHEMOKINE_SIGNALING_PATHWAY
KEGG_MELANOGENESIS
KEGG_GNRH_SIGNALING_PATHWAY
KEGG_GLYCOSPHINGOLIPID_BIOSYNTHESIS_GANGLIO_SERIES
KEGG_T_CELL_RECEPTOR_SIGNALING_PATHWAY
KEGG_THYROID_CANCER

KEGG Positively Enriched in both Olaparib and Talazoparib
KEGG_METABOLISM_OF_XENOBIOTICS_BY_CYTOCHROME_P450
KEGG_DRUG_METABOLISM_CYTOCHROME_P450
KEGG_FATTY_ACID_METABOLISM
KEGG_LINOLEIC_ACID_METABOLISM
KEGG_ARACHIDONIC_ACID_METABOLISM
KEGG_ARGININE_AND_PROLINE_METABOLISM
KEGG_BUTANOATE_METABOLISM
KEGG_STEROID_HORMONE_BIOSYNTHESIS
KEGG_LYSOSOME
KEGG_P53_SIGNALING_PATHWAY
KEGG_CHEMOKINE_SIGNALING_PATHWAY
KEGG_FC_GAMMA_R_MEDIATED_PHAGOCYTOSIS

KEGG Positively Enriched in both Abi+Ola and Enz+Tala
KEGG_TASTE_TRANSDUCTION
KEGG_AXON_GUIDANCE
KEGG_CELL_ADHESION_MOLECULES_CAMS
KEGG_GRAFT_VERSUS_HOST_DISEASE
KEGG_LEUKOCYTE_TRANSENDOTHELIAL_MIGRATION
KEGG_FOCAL_ADHESION
KEGG_ECM_RECEPTOR_INTERACTION
KEGG_NATURAL_KILLER_CELL_MEDIATED_CYTOTOXICITY
KEGG_ERBB_SIGNALING_PATHWAY
KEGG_P53_SIGNALING_PATHWAY
KEGG_LEISHMANIA_INFECTION
KEGG_T_CELL_RECEPTOR_SIGNALING_PATHWAY
KEGG_FC_GAMMA_R_MEDIATED_PHAGOCYTOSIS

KEGG Negatively Enriched in both Abiraterone and Enzalutamide
KEGG_AMINO_SUGAR_AND_NUCLEOTIDE_SUGAR_METABOLISM
KEGG_AMINOACYL_TRNA_BIOSYNTHESIS
KEGG_PROTEIN_EXPORT
KEGG_FRUCTOSE_AND_MANNOSE_METABOLISM
KEGG_SPLICEOSOME
KEGG_TYROSINE_METABOLISM

KEGG Negatively Enriched in both Olaparib and Talazoparib
KEGG_DNA_REPLICATION
KEGG_SPLICEOSOME
KEGG_CELL_CYCLE

KEGG Negatively Enriched in both Abi+Ola and Enz+Tala
KEGG_AMINOACYL_TRNA_BIOSYNTHESIS
KEGG_SPLICEOSOME
KEGG_PROTEIN_EXPORT
KEGG_AMINO_SUGAR_AND_NUCLEOTIDE_SUGAR_METABOLISM
KEGG_PROTEASOME
KEGG_DNA_REPLICATION
KEGG_FRUCTOSE_AND_MANNOSE_METABOLISM
KEGG_CITRATE_CYCLE (TCA cycle)
KEGG_CELL_CYCLE

Supplemental Figure S2: KEGG gene set enrichment analysis (GSEA). KEGG gene sets were investigated by GSEA in samples by groups; ARPi = abiraterone and enzalutamide, PARPi = olaparib and talazoparib, combination = abiraterone + olaparib and enzalutamide + talazoparib. Displayed are gene sets which are both significantly ($p < 0.05$) and either commonly up- or downregulated by group.

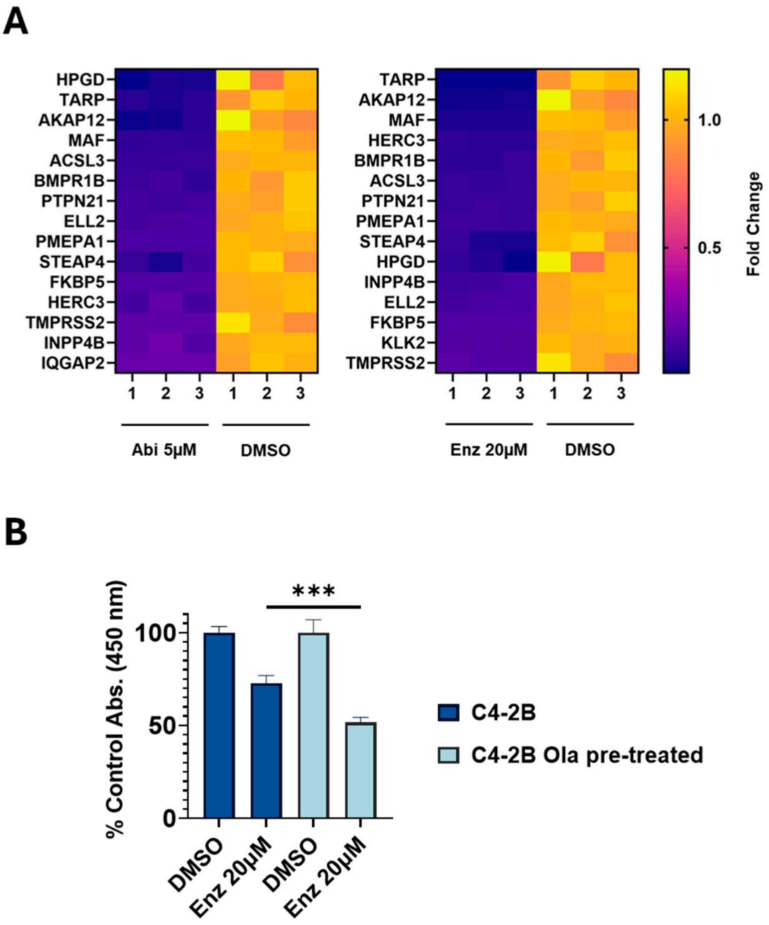

Supplemental Figure S3: Insights into hypothesis that PARP inhibition augments AR transcriptional activity. A. Heatmaps display GSEA hallmark androgen response genes at the leading edge in response to abiraterone or enzalutamide indicating genes most downregulated by treatment in C4-2B cells. **B.** C4-2B cells were pre-treated with olaparib (5µM) for 9 days and subsequently re-plated and tested for enzalutamide sensitivity compared to parental C4-2B cells. Viability was determined after 5-days of enzalutamide treatment via CCK-8. Data is presented as a % of control viability +/- standard deviation (n=4 per condition). Data was analyzed using unpaired two-tailed t-test. *** = p-value ≤ 0.001. Data is representative of three independent experiments.

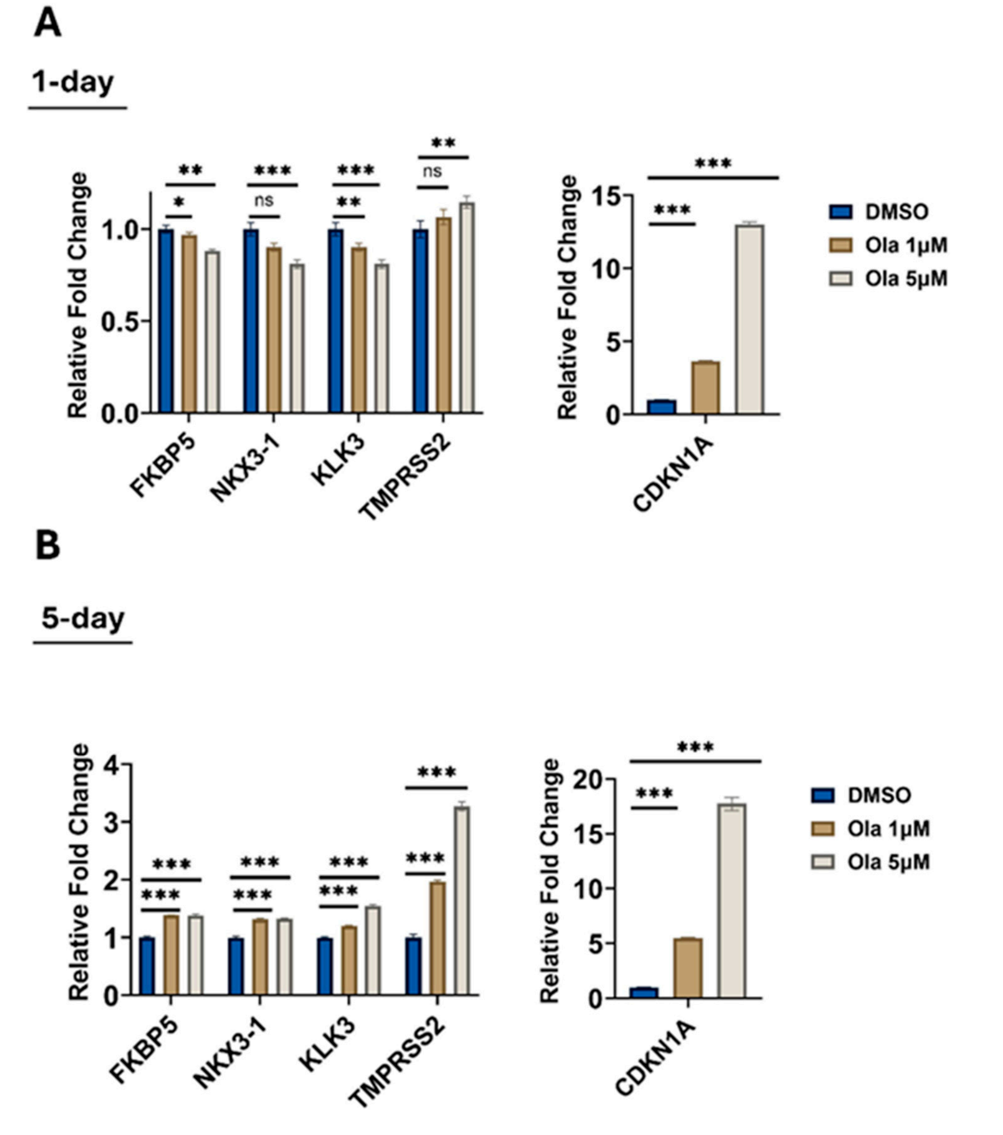

Supplemental Figure S4: Assessing impact of olaparib treatment duration and intensity on AR-target gene expression. C4-2B cells were treated with either 1μM or 5μM olaparib for **A.** 1 or **B.** 5 days. Analysis of RNA-seq read counts (averages of groups normalized to DMSO) for indicated AR-target genes is displayed. CDKN1A (p21) served as a positive control for olaparib treatment. Data is presented as a % of control viability +/- standard deviation (n=3). Data was analyzed using ordinary one-way ANOVA followed by Dunnett's multiple comparison, * = p-value ≤ 0.05, ** = p-value ≤ 0.01, *** = p-value ≤ 0.001, ns = not significant.

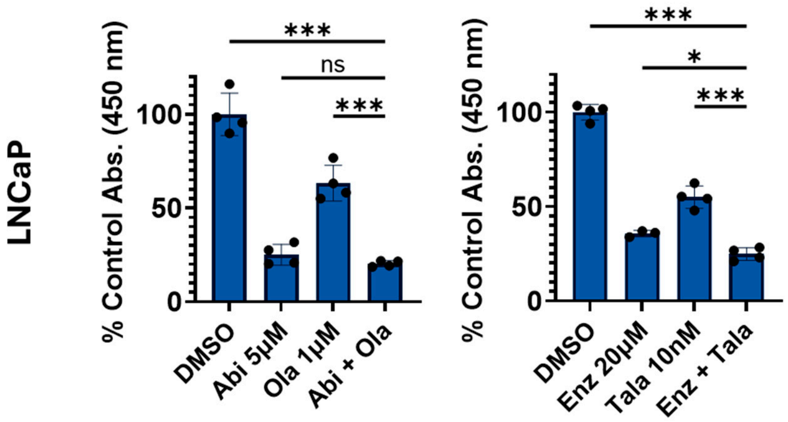

Supplemental Figure S5: PARPi and ARPi combination efficacy testing in LNCaP cells. Viability assays tested response to indicated 5-day treatments in LNCaP cells via CCK-8. Data is presented as a % of control viability +/- standard deviation (n=4, unless otherwise indicated). Data was analyzed using ordinary one-way ANOVA followed by Dunnett's multiple comparison test. Outliers were identified and removed via Grubb's test (α=0.05). One outlier was removed from the LNCaP Enz 20μM condition (right graph). * = p-value ≤ 0.05, *** = p-value ≤ 0.001, ns = not significant. Data is representative of three independent experiments.
